# Nanoparticle-mediated selective Sfrp-1 silencing enhances bone density in osteoporotic mice

**DOI:** 10.1186/s12951-022-01674-5

**Published:** 2022-10-29

**Authors:** Patricia García-García, Ricardo Reyes, Daniel García-Sánchez, Flor María Pérez-Campo, José Carlos Rodríguez-Rey, Carmen Évora, Patricia Díaz-Rodríguez, Araceli Delgado

**Affiliations:** 1 Department of Chemical Engineering and Pharmaceutical Technology, Universidad de La Laguna, 38206 La Laguna, Spain; 2 Institute of Biomedical Technologies (ITB), Universidad de La Laguna, 38320 La Laguna, Spain; 3 Department of Biochemistry, Microbiology, Cell Biology and Genetics, Universidad de La Laguna, 38206 La Laguna, Spain; 4 Department of Molecular Biology, Faculty of Medicine, University of Cantabria-IDIVAL, 39012 Santander, Spain; 5 Department of Pharmacology, Pharmacy and Pharmaceutical Technology, I+D Farma Group (GI-1645), Facultad de Farmacia, Instituto de Materiales (iMATUS) and Health Research Institute of Santiago de Compostela (IDIS), Universidade de Santiago de Compostela, 15782 Santiago de Compostela, Spain

**Keywords:** Bone regeneration, Osteoporosis, Gene therapy, Lipid-polymer hybrid nanoparticles

## Abstract

Osteoporosis (OP) is characterized by a loss in bone mass and mineral density. The stimulation of the canonical Wnt/β-catenin pathway has been reported to promote bone formation, this pathway is controlled by several regulators as secreted frizzled-related protein-1 (Sfrp-1), antagonist of the pathway. Thus, Sfrp-1 silencing therapies could be suitable for enhancing bone growth. However, the systemic stimulation of Wnt/β-catenin has been correlated with side effects. This work hypothesizes the administration of lipid-polymer NPs (LPNPs) functionalized with a MSC specific aptamer (Apt) and carrying a SFRP1 silencing GapmeR, could favor bone formation in OP with minimal undesired effects. Suitable SFRP1 GapmeR-loaded Apt-LPNPs (Apt-LPNPs-SFRP1) were administered in osteoporotic mice and their biodistribution, toxicity and bone induction capacity were evaluated. The aptamer functionalization of the NPs modified their biodistribution profile showing a four-fold increase in the bone accumulation and a ten-fold decrease in the hepatic accumulation compared to naked LPNPs. Moreover, the histological evaluation revealed evident changes in bone structure observing a more compact trabecular bone and a cortical bone thickness increase in the Apt-LPNPs-SFRP1 treated mice with no toxic effects. Therefore, these LPNPs showed suitable properties and biodistribution profiles leading to an enhancement on the bone density of osteoporotic mice.

**Supplementary Information:**

The online version contains supplementary material available at 10.1186/s12951-022-01674-5.

## Introduction

Osteoporosis is recognized as a global public health problem representing a heavy socioeconomic burden systemic disease. This pathology is characterized by a loss in bone mass and Bone Mineral Density (BMD) [1]. The increased longevity of the population, in both developed and developing countries, has led to a global demographic change and, therefore, new challenges for the healthcare systems [2]. The normal process of aging entails the loss of bone that can culminate in osteoporosis, representing a huge threat to the quality of life for elderly people. Therefore, new strategies for the development of more efficient and with lower side-effect osteoporosis treatments are intensively being searched.

The stimulation of the canonical Wnt/β-catenin pathway promotes bone formation by favoring the differentiation of precursor cells into osteoblasts while inhibiting their conversion into adipocytes [3]. This pathway is controlled by several extracellular and intracellular proteins. Among them, the soluble secreted frizzled-related protein 1, Sfrp-1 acts as Wnt antagonist, this protein contains a cysteine-rich domain in its amino terminus that sequesters Wnts and prevents their binding to the Frizzled receptors resulting in the inhibition of the pathway [4]. Sfrp-1-defective mice show increased trabecular bone formation. Moreover, in mice calvaria, the inhibition of Sfrp-1, using WAY-316606-based molecules, resulted in an increased bone formation [5]. Using GapmeRs, chimeric antisense oligonucleotides, to inhibit Sfrp-1 expression, our group has shown increased osteoblastic differentiation of MSCs in culture, including human cells obtained from osteoporotic patients. Furthermore, our above mentioned studies indicated the inhibition of Sfrp-1 in cultured MSCs does not appear to increase the risk of uncontrolled cell proliferation but led to an increased bone production in an ectopic mice model [6]. Thus, the development of Sfrp-1 silencing based therapies could be a viable strategy for enhancing bone formation when diminished bone growth is present such as primary osteoporosis and glucocorticoid induced osteoporosis [7–9].

Preclinical and clinical studies of the anti-sclerostin antibody Romozosumab, suggest the activation of the canonical Wnt signaling does not increase the carcinogenic risk [10]. However, there are reports of major cardiovascular events in Romozosumab patients and, also in aged Sfrp-1-defective mice [11]. Thus, the development of Wnt signaling activators with an effect restricted to bone precursor cells is of great interest. The use of nanoparticles (NPs) and/or nanocomplexes for targeted delivery of nucleic acids to the affected tissues could be useful to avoid undesirable systemic effects while ensuring the stability of the cargo.

Aptamers (Apt) are small, single-stranded nucleic acid molecules or peptides able to recognize and bind specific structures based on their three-dimensional conformation with similar affinities to those of antibodies [12, 13]. Because of their small size, they have lower immunogenicity, and, since their three-dimensional structure is uniquely sequence-dependent, batch-to-batch variability is negligible. Aptamers can even be selected against whole cells by a so-called cell-selex procedure [14]. These molecules have been used as targeting ligands in diagnosis and therapy, either directly conjugated to oligonucleotides or included into the nanomaterials’ structures [15–18]. The application of these aptamer-functionalized drug nanosystems has been mainly focused on the treatment of cancer [16, 19, 20]. However, there are other pathologies such as osteoporosis that could be highly beneficiated by this strategy. In this sense, the intra-bone marrow injection of an antagomiR-188 PEI-citrate complex including an MSC-specific aptamer resulted in a bone mass increase [21]. Also, the delivery of a *Plekho1* siRNA to osteoblasts by (AspSerSer)_6_ or CH6 aptamer-functionalized lipid NPs resulted in an increase in bone formation [22, 23]. These data indicate the feasibility of the aptamer-targeted silencing approaches for bone related disorders.

Although PEI has been one of the most widely used cationic polymers for nucleic acid condensation, its toxicity, related to its molecular weight hinders their therapeutical use [24]. Liposomes are also traditionally used as non-viral gene delivery systems however, their instability and weak structural integrity limit their use [25]. Alternatively, lipid-polymer NPs (LPNPs) have emerged to overcome these limitations, where the polymeric core offers high cargo protection, and the lipid or lipid-PEG corona provides biomimetic properties and stealth NPs reducing opsonization [26]. Still, LPNPs have been little explored as nanocarriers for bone targeting, relegating their use to the treatment of osteosarcoma [27, 28].

This work hypothesizes the systemic administration of LPNPs functionalized with a MSC-specific Apt and carrying an SFRP1 silencing GapmeR, antagonist of the Wnt/β-catenin signaling pathway, could promote the increase of the osteogenic potential of MSCs, favoring the formation of new bone tissue in OP. To this end, LPNPs composed of a PLGA core, encapsulating the GapmeR, surrounded by a lipid layer of DOTAP, L-α-phosphatidylcholine, DSPE-mPEG2000 and DSPE-PEG2000-maleimide were designed. The lipid-Maleimide allow the covalent aptamer-NP binding [29] so this approach offers a nanoparticle interface with: (1) PEG to reduce opsonization and (2) Aptamer to target the nanocarrier to the osteoprogenitor cells. The effect of pH, GapmeR encapsulation and Apt functionalization on the LPNPs physicochemical properties was evaluated. Also, the oligonucleotide encapsulation efficiency and in vitro release at variable temperatures was studied. Adequate SFRP1 GapmeR-loaded Apt-LPNPs were then evaluated in terms of cytocompatibility, cell uptake, intracellular localization, and gene silencing efficiency. Finally, the developed systems were systemically administered via tail vain in vivo in an mice osteoporotic model and their biodistribution, toxicity and bone induction capacity were evaluated.

## Materials and methods

### Reagents

Poly(D,L-lactide-co-glycolide) (Resomer^®^ RG502H, Mw 7000–17,000) was purchased from Evonik (Germany), Soy L-α-phosphatidylcholine (95%) (PC) was obtained from Avanti Polar Lipids (USA), DSPE-mPEG(2000) (1,2-distearoyl-sn-glycero-3-phosphoethanolamine-N-[methoxy(polyethylene glycol)] and DSPE-PEG(2000)-Maleimide (1,2-distearoyl-sn-glycero-3-phosphoethanolamine-N-[maleimide(polyethylene glycol)-2000] were provided by Nanosoft Polymers (USA). DOTAP (1,2-dioleoyl-3-trimethylammonium-propane) (Mw 774.19), protamine sulfate (Mw 5000–10,000), Tris (2-carboxyethyl) phosphine hydrochloride (TCEP) (Mw 286.65) and coumarin-6 (Mw 350.25) were purchased from Sigma-Aldrich (USA). The specific GapmeR to silence the gene expression of SFRP1 (5′-GGTCAGTAACTAAGTT- 3′) and the control GapmeR without therapeutical activity (5′- AACACGTCTATACGC- 3′) labelled or not at 5′ with FAM were purchased and designed by Quiagen (Germany). The specific aptamer for murine bone marrow mesenchymal stem cells (mMSC) (5′-GAATTCAGTCGGACAGCGACGACGGTGATATGTCAAGGTCGTATGCACGAGTCAGAGGGATGGACGAATATCGTCTCCC-3′) was provided by Integrated DNA Technologies (USA).

### Lipid-polymer hybrid nanoparticles synthesis and characterization

#### Synthesis procedure: modified nanoprecipitation method

Different LPNPs formulations were prepared to test the pH effect on the LPNPs physicochemical properties. LPNPs of variable shell composition were obtained by modifying their lipid components while keeping all the remaining variables constant leading to four LPNPs formulations with the following lipid composition: (1) DOTAP; (2) DSPE-mPEG2000; (3) PC + DSPE-mPEG2000 and (4) DOTAP + PC + DSPE-mPEG2000. LPNPs were developed by a modified single-step nanoprecipitation method similarly to previously described [30]. Briefly, 1.32 µg of GapmeR (26.4 µg/mL) was incubated with protamine sulfate (0.9 mg/mL) for 40 min at room temperature before NPs preparation. Then, 50 µL of the GapmeR + protamine solution was added to the organic phase formed by PLGA (5 mg/mL) or PLGA (5 mg/mL) + DOTAP (0.3 mg/mL) when required in 1 mL of acetone and mixed. This mixture was poured in 10 mL of 4% EtOH in water containing DSPE-mPEG2000 (0.0637 mg/mL) alone or with PC (0.02 mg/mL) previously dissolved at 65 °C [31] (aqueous phase). NPs were kept under constant magnetic stirring for 1 h to evaporate the solvent. After solvent removal, LPNPs were concentrated to a final volume of 400 µL by ultrafiltration using Amicon filters (Amicon^®^Ultra 100 kDa, Merck Millipore, Ireland) at 10,000 rpm for 10 min. The obtained nanoparticles were characterized at native pH in water and diluted in phosphate buffer at pH 5.4, 6.0, 7.0 or 7.4.

For aptamer conjugation, DSPE-PEG2000-maleimide was added to the aqueous phase replacing part of the DSPE-mPEG2000 as previously shown [29]. On the other hand, fluorescently labelled nanoparticles were obtained by adding coumarin-6 at 4 µg/mL to the organic phase.

#### Dynamic light scattering (DLS)

All LPNPs batches were characterized in terms of average diameter, polydispersity index (PdI), and zeta-potential (ζ-potential) by dynamic light scattering using a Zetasizer Nano (Malvern). All measurements were made in triplicate. Before characterization, samples were appropriately diluted with MilliQ-water (1:10) and sonicated in a water bath for 1 min.

#### Transmission electron microscopy (TEM)

LPNPs were morphologically characterized by transmission electron microscopy (TEM). Samples (10 µL) were deposited on carbon membrane coated cooper grids and stained with a 2% w/v phosphotungstic acid solution for 2 min. LPNPs morphology was assessed using the JEM 2010 electron microscope (JEOL, Japan).

### LPNPs surface modification using a murine mesenchymal stem cells specific aptamer

The surface functionalization of LPNPs with a murine mesenchymal stem cells (mMSC) specific aptamer was performed as already reported [29]. Briefly, NPs suspensions obtained as described in "Synthesis procedure: modified nanoprecipitation method" section. were buffered with 5X phosphate buffer to get a final pH of 7.4 and incubated with thiol-reactive aptamer for 1 h at room temperature. To obtain the thiol-reactive aptamer the commercial SS-modified aptamer was reduced to aptamer-SH by its incubation with a 100-fold excess of (tris(2-carboxyethyl)phosphine) (TCEP) for 2 h at room temperature. The final aptamer:maleimide molar ratio used for reaction was 0.01:1. Functionalized LPNPs were characterized in terms of average diameter, zeta-potential and PdI.

The aptamer conjugation efficiency was measured using FAM-modified aptamer. The non-associated aptamer was quantified after NPs ultrafiltration (Amicon^®^Ultra filters 100 kDa, 10.000 rpm for 10 min) using a plate reader (Biotek, USA) at 485/528 nm.

### Oligonucleotide encapsulation efficiency and release

Oligonucleotide encapsulation efficiency (EE) and release profile were evaluated using fluorescently labelled GapmeR. To assess the NPs EE, the non-encapsulated GapmeR was quantified at 485/528 nm after LPNPs preparation and ultrafiltration as described above. The amount of GapmeR released during aptamer conjugation was also measured following the same procedure.

$$EE \left(\%\right)= \frac{Total\, GapmeR-Free\, GapmeR}{Total\, GapmeR} \times 100,$$

where free GapmeR is the amount of the non-incorporated oligonucleotide during LPNPs preparation or functionalization procedures, respectively.

Furthermore, in vitro GapmeR release assays were carried out diluting the loaded LPNPs with DEPC water and incubating the resultant suspensions at 4 °C or 37 °C. At different time points, samples were ultrafiltrated (Amicon^®^Ultra filters 100 kDa, 10,000 rpm for 10 min) and the free GapmeR was quantified at 485/528 nm in the filtrated solution. The assay was carried out by triplicate.

### Nanoparticle’s cytotoxicity, cell uptake and intracellular location

LPNPs cytotoxicity was studied in primary mMSC and macrophages (Raw 264.7 ATTC, USA). mMSC were isolated from FVB mice as previously described [32]. Cells were seeded at 20,000 cells per well on 96-well plates with complete culture media (DMEM + 10% Bovine Fetal Serum (FBS), 1% Penicillin/streptomycin and 1% L-Glutamine) and allowed to attach for 24 h. Afterwards, cell monolayers were incubated for 24 h with naked or aptamer-functionalized LPNPs suspensions in DMEM at variable concentrations (0.1 µg/mL–1.41 mg/mL). Cell viability was measured by an XTT assay (Roche) following the manufacturer’s instructions. All tests were carried out by triplicate.

To assess LPNPs cell uptake, fluorescently labelled nanoparticles were used. mMSCs were seeded at the same density as described above for the cytotoxicity tests in 24-well plates and incubated with the different nanoparticles’ suspensions for 2 h. Then, the medium was removed, the cells were washed twice with DPBS and tripsinized at 37 °C and 5% CO_2_ for 5 min, the cells suspension was then centrifugated at 2100 rpm for 4 min and cells were analyzed with the Tali^®^ Image-Based Cytometer (Thermofisher). The percentage of cell uptake was obtained by counting both the cells stained with coumarin-6 and total cells. The ability of the aptamer conjugation to specifically enhance NPs uptake in MSCs was also assessed. To this end, NPs uptake was quantified as previously published by our group in murine MSC cell line C3H10T1/2 and fibroblasts (BALB/3T3 clone A31, ATTC, USA) cultured in the above mentioned complete culture media [33]. Briefly, cells were seeded at the same density as previous experiments in 96-well plates and allowed to attach for 18 h. Afterwards, NPs suspensions were added to each well and fluorescence was measured using a microplate reader (Bio Tek). Cell monolayers were then incubated for 2 h with the NPs and subsequently washed three times with DPBS to remove non-internalized NPs. Lastly, cells were lysed with Triton X-100 1% and the fluorescence of the lysates was measured. Relative NPs uptake was obtained dividing the blank corrected lysates fluorescence by the blank corrected initial fluorescence. Alternatively, NPs uptake was visualized by fluorescence microscopy and transmission electron microscopy. For the fluorescence microscopy cells were seeded at the same density as described above in a culture slide (Falcon^®^, CultureSlides) and treated with the LPNPs suspensions for 2 h, washed twice with DPBS and fixed with 4% paraformaldehyde for 2 h at room temperature. Afterwards, cells were washed twice with DPBS and permeabilized with 0.1% Triton X-100 for 15 min, followed by two washes with DPBS and an incubation with 50 µg/mL rhodamine phalloidin (Thermo Fisher Scientific, USA) for 40 min. Then, wells were washed twice and incubated with 1 µg/mL of DAPI for 5 min. Finally, cells were visualized using a fluorescence microscope EVOS M5000 Imaging System (ThermoFisher). The internalization pathway followed by the LPNPs was evaluated by transmission electron microscopy. Cells were seeded at the same density as previously described on Petri dishes and treated for 2 h with the nanoparticle’s suspensions. Cells were fixed in 3% glutaraldehyde in phosphate buffer (0.12 M Na_2_HPO_4_ and 0.12 M NaH_2_PO_4_; pH 7.2) for 60 min at room temperature. Then they were scraped off from the dishes, transferred to a 1.5 mL tube, and sequentially centrifuged at 7000 rpm and 12,000 rpm for 10 min. Carefully, samples were washed with 0.12 M phosphate buffer without disturbing the pellet, and further fixed in 1% OsO_4_ for three hours under mild agitation. The cell pellet was dehydrated in increased acetone concentrations and embedded in Araldite (Durcupan, Fluka, Switzerland). Ultrathin sections mounted in copper grids were stained with lead citrate and uranyl acetate and examined with a JEOL 2011 electron microscope operated at 80 kV.

### In vitro NPs gene silencing efficiency using primary mMSC and immortalized cell lines (C3H10T1/2)

The in vitro SFRP1 silencing efficiency of GapmeR-loaded NPs functionalized or not with an mMSC-specific aptamer was assessed using both murine primary MSC (mMSC) and an the immortalized murine MSC line C3H10T1/2. Cells were cultured with complete culture media (DMEM + 10% FBS + 1% P/S) and tripsinized when reaching a confluence of 70%. Primary cells were used at passage 2. Cells were seeded on 24-well plates at a density of 25,000 cells per well and let to attach overnight. Two hours before the transfection, cell culture media was removed and cell monolayers were washed with DPBS (Lonza) followed by the addition of the correspondent treatments in basal cell culture media (Opti-MEM, Life Technologies Inc., Gaithersburg, MD) without supplementation. Cell monolayers were treated with 400 µL of the following conditions: (1) No treatment (basal media); (2) Control GapmeR previously complexed with the transfection reagent Dharmafect (20 nM) (Cntrl); (3) Control GapmeR encapsulated in unfuntionalized NPs (equivalent GapmeR concentration 20 nM) (LPNPs-Cntrl); (4) SFRP1 silencing GapmeR previously complexed with the transfection reagent Dharmafect (20 nM) (SFRP1); (5) SFRP1 silencing GapmeR encapsulated in unfuntionalized NPs (equivalent GapmeR concentration 20 nM) (LPNPs-SFRP1) and (6) SFRP1 silencing GapmeR encapsulated in aptamer funtionalized NPs (equivalent GapmeR concentration 20 nM) (Apt-LPNPs-SFRP1) cells were kept in standard cell culture conditions (37 °C, 5% CO_2_). GapmeR-Dharmafect complexes were obtained following standard protocols [Dharmafect (Dharmacon, Horizon Discovery, Cambridge, UK)]. Briefly, both GapmeR and Dharmafect were diluted to the desired concentration in basal cell culture media and incubated 5 min at room temperature. Afterwards, the GapmeR solution was drop-wise added to the Dharmafect solution and the mixture was allowed to settle for 20 min at room temperature then, complexes were added to the cell monolayers.

After 24 h of treatment an equal volume of complete media was added to the wells and at 48 h post-treatment mRNA was isolated from cell cultures. To this end, cells were washed twice with PBS and collected with Trizol by scrapping. RNA was extracted by the phenol–chloroform technique. RNA reverse transcription was carried out with the PrimeScript RT reagent kit (RR037A, Takara Bio Inc, Shiga, Japan) according to the manufacturer’s protocol. For the gene expression measurements, a real time quantitative PCR was performed using Taqman assay (Thermo Fisher Scientific).

Gene silencing was tested using Mm00489161_m1 (Sfrp1) Taqman assay. Housekeeping genes *GAPDH* (Assay Mm99999915_G1) and *RPL13A* (Assay Mm0162986_gH) were used for normalization.

### LPNPs biodistribution

Animal experiments were performed according to the European Union legislation on Care and Use of Animals in Experimental Procedures (2010/63/UE) and after approval by the Ethic Committee for animal care of University of La Laguna (CEIBA2018-0310).

Biodistribution experiments were carried out with both naked LPNPs (^99m^Tc-LPNPs) and LPNPs functionalized with the aptamer (^99m^Tc-Apt-LPNPs) using two groups of 12 animals. The nanoparticle suspension (50 µL at 3 mg/mL) was injected in the tail vein of each mice under fasted conditions. At prefixed times (1, 4 and 14 h) 4 mice of each group were euthanized. Blood samples were collected (by cardiac puncture) and the heart, liver, lungs, kidneys, spleen, thyroid, brain, femur, tibia, hip, ribs and skull, were removed. Organ and blood associated activity was counted using a gamma counter (Packard, Cobra II). Results are expressed as the percentage of the total administered dose per gram of tissue.

LPNPs were labelled with ^99m^Tc using SnCl_2_ H_2_O as reducing agent and ascorbic acid as antioxidant, under nitrogen atmosphere. The labelling was carried out adding 1–1.5 mCi of ^99m^TcO_4_Na in saline solution to 0.85 mL of the LPNPs suspension (3.6 mg/mL) [34]. The suspension was shaken for 10 min at 150 rpm and the labelled suspension was buffered to a pH of 7.2–7.4 with 150 µL of NaHCO_3_ (0.5 M). The labelling efficiency was assessed by instant thin layer chromatography (iTLC) using silica gel-coated strips (Varian Iberica, S.L.) with acetone as the mobile phase. The labelling efficiency was quantified by counting the radioactivity in the two equal halves of the strip using a gamma counter (Packard, Cobra II). Free ^99m^TcO_4_^−^ migrates to the front while radiolabeled LPNPs remained at the dripped point. Radiolabeling efficiency was also confirmed by filtration using Amicon centrifugal filters (Amicon®Ultra filters MWCO 100 kDa). Free radioactivity in the filtrate was measured. The radiolabeling stability of the formulations was checked over a period of 24 h by iTLC, as described above.

### In vivo* osteoinduction capacity of Apt-LPNPs-SRFP1*

To evaluate the in vivo effect of the developed LPNPs in the bone tissue of osteoporotic mice 18 female 16-week-old FVB mice were used in this study. The osteoporosis model was performed as previously described [32] by bilateral ovariectomy (OVX) combined with subcutaneous administration of 3 mg/kg dexamethasone 21-isonicotinata (DEX) (Deyanil Retard, Fatro Ibérica, Spain) every week for three months. The osteoporotic-like bone condition was previously validated [32].

After three months, animals were divided in three groups and treated with 50 µL per month by intravenous administration via tail with one of the following treatments: (1) physiological saline solution (Control); (2) Apt-LPNPs-Cntrl 3 mg/mL or (3) Apt-LPNPs-SFRP1 3 mg/mL. The stablished GapmeR dose was in all cases 25 ng. Animals were monitored by densitometry (PIXImus, GE Lunar, USA) under isofluorane anesthesia the day before each dose administration and euthanasia (0, 1, 2 and 3 months) [32]. At three months, mice were euthanized by perfusion fixation under anesthesia [35] and bones and organs were extracted for histological and immunohistochemistry assessments.

#### Histological and histomorphometric and analyses

To determine the effect of gene silencing on osteoporotic bone structure and microarchitecture, the femurs were extracted and prepared for histological analysis as previously described [36]. Briefly, samples were fixed in 4% paraformaldehyde solution, decalcified in Histofix^®^ Decalcifier (Panreac, Barcelona, Spain) and embedded in Paraplast^®^. Longitudinal sections of 5 µm thick were obtained from each of the femurs with a microtome (Shandon Finesse 325). The sections were stained with hematoxylin-erythrosine for bone structure evaluation. While bone mineralization was assessed with VOF trichrome stain, in which red-brown staining indicates advanced mineralization, whereas less mineralized, newly formed bone stains blue [37]. The histomorphometric analysis was carried out by measuring the following parameters: thickness of the cortical bone (Ct.Wi) and number (Tb.N), width (Tb.Wi) and separation (Tb.Sp) of the trabeculae in trabecular bone. Sections were analyzed by light microscopy (LEICA DM 4000B) and computer-based image analysis software (Leica Q-win V3 Pro-Image Analysis System, Barcelona, Spain) was used to evaluate the above mentioned histomorphometrical parameters. In this study, the histomorphometric parameters have been evaluated in 2D as direct indexes, using 5 µm thick longitudinal sections throughout the entire femur. Between 8 and 12 sections per animal were evaluated. The number of trabeculae (Tb.N) was determined, considering different trabeculae those in different directions or orientations. Trabecular separation (Tb.Sp), defined as the distance between the borders of the trabeculae. Trabecular thickness (Tb.Wi) and cortical bone thickness (Ct.Wi), as the distance between the edges in each of these structures, measured at different points along them. The measurements of the structural parameters of the cancellous bone (Tb.N, Tb.Sp and Tb.Wi) were made in cancellous bone of both epiphyseal regions and, the structural measurements of the cortical bone (Ct.Wi) were made in both, epiphyseal and diaphyseal bone regions.

#### Immunohistochemistry analyses

The expression of collagen type I (Col I), an early osteogenesis marker, and osteocalcin (OCN), a late osteogenesis and mineralization marker, were evaluated by immunohistochemical analysis. To this end, sections were deparaffined and rehydrated in Tris-buffered saline (TBS) (pH 7.4, 0.01 M Trizma base, 0.04 M Tris hydrochloride, 0.15 M NaCl), which was used for all further incubations and rinse steps. Sections were incubated in citrate buffer (pH 6) at 90 °C for antigen retrieval during 5 min. After a rinse step, sections were blocked with 2% FBS in TBS–0.2% Triton X-100 (blocking buffer). The indirect immunohistochemical procedure was carried out by incubating the sections with collagen type I (Col I) and osteocalcin (OCN) polyclonal antiserum (1/100) (Millipore, Barcelona, Spain) in blocking buffer overnight at 4 °C. Sections were rinsed three times, then incubated with Cy3 donkey anti-rabbit F(ab) fragment (1/500) (Millipore, Barcelona, Spain) in blocking buffer for 1 h. After two rinse steps, the sections were mounted with buffered glycerin. The analysis of the samples was carried out in an optical microscope (Leica DM4000B) with a fluorescence lamp, and the images were captured with a digital camera (Leica DFC300FX). Reaction specificity was confirmed by replacing the specific antiserum with normal serum, and an autofluorescence control was also performed omitting both antisera.

Col I and OCN staining was measured using computer-based image analysis software (ImageJ, NIH, Bethesda, MD) by applying a fixed threshold to select for positive staining in different bone regions of the femur, both epiphyseal regions and in the diaphysis. Positive pixel area was divided by the total surface bone size. Values were normalized to those measured from Apt-LPNPs-Cntrl group and reported as relative staining intensities.

#### Toxicity

To analyze the organs toxicity spleen, liver, lung, kidney, heart, and brain were extracted and prepared for histological analysis as previously described [36]. Briefly, the organs were post fixed by immersion for 24 h in the same fixative and embedded in Paraplast^®^. Sections of 5 µm thick were obtained, stained with hematoxylin-erythrosine and Cleveland-Rucker-Wolfe for topographical study and analyzed by light microscopy.

### Statistical analysis

Statistical analysis was performed using SPSS 21.0 software. First, the normality and homoscedasticity of the data were checked using the Kolmogorov–Smirnov test and the Levene test, respectively. This was followed by a one-way analysis of variance (ANOVA I) with a post-test of Tukey's multiple comparison. The results are expressed as mean ± standard error of the mean (SEM) and statistically significant differences are considered at *p-values* < 0.05.

## Results

### LPNPs physicochemical properties

**Fig. 1:**
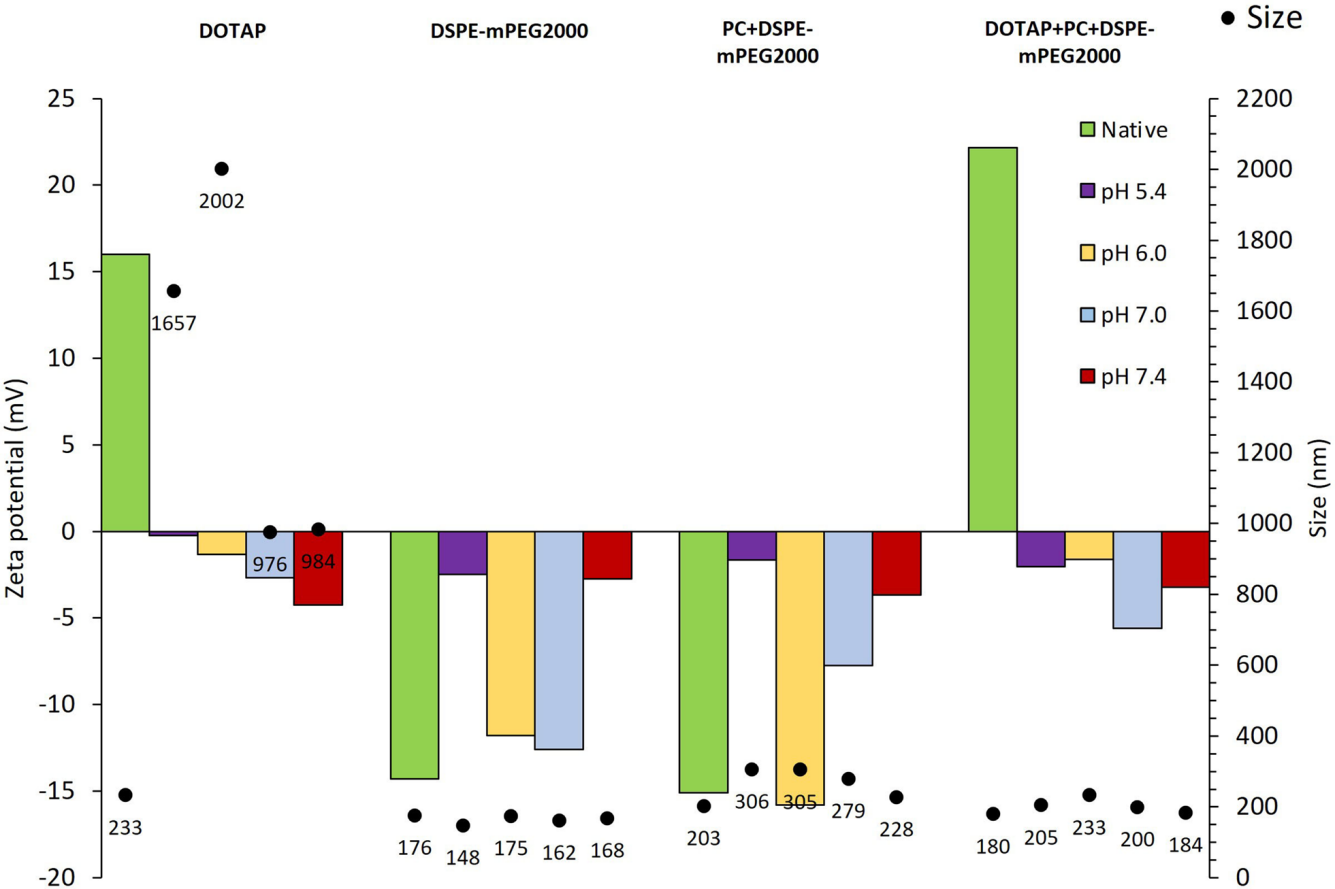
LPNPs properties in phosphate buffer at different pHs 5.4, 6.0, 7.0, 7.4 or in water at native conditions. Histogram indicates the Zeta potential (mV) values at different conditions and black points indicates nanoparticle size (nm) (n = 3)

Despite of being synthesized in water nanoparticles are subjected to different environments of higher ionic content when administered. It is widely stablished that endocytosis is a usual path for cell nanoparticle uptake [38]. During this procedure nanomaterials are exposed to variable conditions, initially held at the early endosome, pH ≈ 6 and then to the late endosome pH ≈ 5.5 once at this point, nanomaterials are either kept inside the endosome as it maturates towards the lysosome formation and their degradation (pH 4.5) or escape to the endosome through different mechanisms and released in the cytosol (pH 7.4) [38–40]. When nanoparticles are used as delivery vehicles of labile molecules aimed at promoting their therapeutic effect at the cell cytosol as, antisense oligonucleotides, endosomal escape is required. Therefore, our nanoparticles are aimed to follow the early endosome pH 6 > late endosome pH 5.5 > cytoplasm pH 7.4 pathway. To mimic this behaviour the developed formulations were subjected to these pHs. Cationic lipids and negatively-charged phospholipids typically located on the cytoplasmic side of the endosomal membrane as phosphatidylserine are recognized to induce endosomal escape [41]. Following this strategy, hybrid DOTAP-PLGA nanoparticles were prepared (DOTAP) of around 200 nm. However, when subjected to higher pHs in phosphate buffer, a dramatic decrease in surface charge and an increase in particle size was observed with unacceptable values of mean diameter of about 1000 nm (Fig. 1). To overcome this drawback phosphatidylcholine (PC) and a pegylated phospholipid (DSPE-mPEG) were added to the formulation. As expected, despite not modifying the drastic change in zeta potential the inclusion of both components increased the stability of the formulation avoiding these undesirable nanoparticle aggregations, showing similar average diameters at all the pHs tested and adequate stability under physiological conditions.

### Optimized LPNPs characteristics

**Fig. 2:**
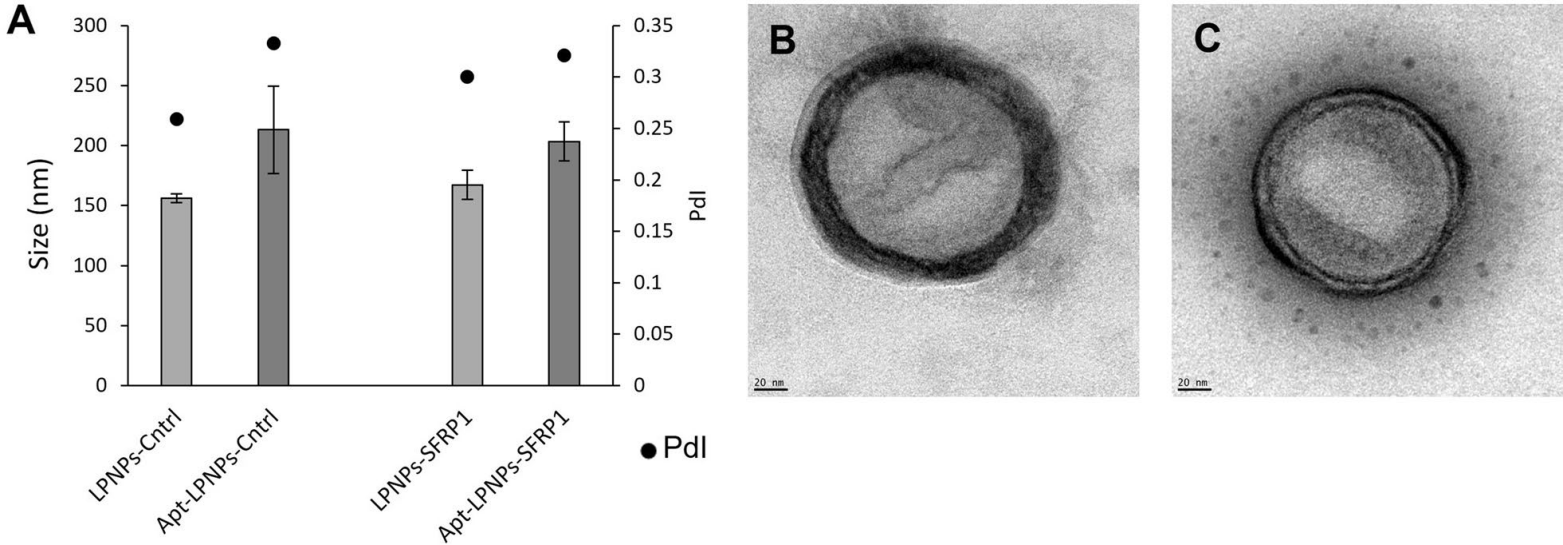
**A** LPNPs-Cntrl and LPNPs-SFRP1 size (nm) and polydispersity index before and after aptamer conjugation obtained by DLS and Transmission electron micrographs of **B** LPNPs-SRFP1 and **C** Apt-LPNPs-SFRP1

Once the most suitable hybrid nanoparticles composition was selected (DOTAP + PC + DSPE-mPEG2000), systems were loaded with both control and SFRP1-silencing oligonucleotides (GapmeRs). As expected, the developed NPs showed an adequate average diameter, of approximately 160 nm (165.6 ± 21.9 nm for control GapmeR and 168.2 ± 22.89 nm for SFRP1-silencing GapmeR), independently of the encapsulated GapmeR. Moreover, these formulations presented low polydispersity indexes (< 0.3) similar to those already reported by our research team for similar hybrid nanoparticles [29]. However, after the aptamer functionalization step, a slight increase in both polydispersity index and average diameter was observed, although not statistically significant, indicative of the aptamer incorporation in the nanoparticles’ surface (Fig. 2A). In agreement with this, the surface charge of the nanoparticles also changed to a similar extend for both formulations, decreasing 18 mV for the LPNPs loaded with control GapmeR and 21 mV for the ones loaded with SFRP1-silencing GapmeR. This behavior may be correlated to the presence of the oligonucleotide, negatively charged, on the nanoparticles surface and was previously observed by our group [29]. On the other hand the TEM images (Fig. 2B and C) show the characteristic core–shell structure of this type of nanoparticles.

The efficiency of the developed LPNPs to encapsulate the oligonucleotide was assessed using a fluorescently labelled GapmeR and stablished as 63.55 ± 4.32%. The GapmeR release was determined at two temperatures 4 °C to stablish the adequate LPNPs suspension storage period and 37 °C to predict, with the known limitations of in vitro tests, the expected in vivo release profile. A biphasic pattern release was observed at both temperatures. However, the lowest temperature seemed to favour the GapmeR retention within the particle matrix, leading to 15.35 and 30.40 release percentages after 16 h and 48 h of storage, respectively. Interestingly, these results suggest the LPNPs suspension could be safely stored in a fridge during several hours without compromising their oligonucleotide payload. Contrarily, a faster GapmeR release occurred at 37 °C, with the 32.85% of the GapmeR released just after 16 h of study and 41.74% by 48 h.

### Nanoparticle’s cytotoxicity and cell uptake

**Fig. 3:**
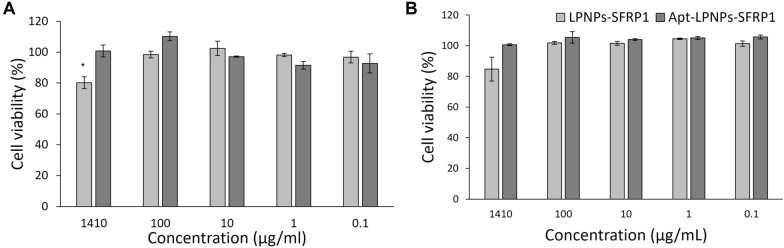
Cell viability of **A** primary murine bone marrow mesenchymal stem cells (mMSC) and **B** murine macrophages (Raw 264.7) after 24 h of treatment with the LPNPs suspensions at variable concentrations (1.41 mg/mL-0.1 µg/mL). (*) denotes statistical significance to control (non-treated cells) p < 0.05 (n = 3)

The treatment of mMSC and macrophages with the different nanoparticles’ concentrations did not show any toxicity at NPs concentrations below 100 µg/ml (Fig. 3). Only a significant decrease in mMSC viability was observed at the highest NPs concentration (1.41 mg/mL) when compared to control (p-value = 0.01) but even in this case, cell viability remained higher than 70%. These results indicate the safety of the developed LPNPs.

Together with cytocompatibility, adequate cell uptake is a desirable characteristic for nanoparticles designed to intracellularly deliver antisense oligonucleotides. Therefore, the NPs uptake by the target cells, mMSC, and the internalization pathway followed in these cells were studied using different techniques. The number of cells positive for coumarin-6 (the used dye to label LPNPs) was assessed using an image-based cytometer. The obtained experimental data showed that 99% of the cells treated with LPNPs were positive for coumarin-6 staining while 100% of the cells treated with Apt-LPNPs were positive for coumarin-6 staining. Therefore, both particles were highly internalized by cells. Despite of not observing differences in the number of positive cells for both types of NPs, the labelling intensity was slightly higher for Apt-LPNP which could indicate a higher number of internalized NPs per cell in this treatment. Additionally, the specificity and percentage of NPs internalized by the cells was also measured (Additional file 1: Fig. S1). These data corroborate a higher number of NPs internalized for Apt-LPNPs than for LPNPs in mMSCs while no effect of the Apt functionalization was observed for fibroblasts indicating the specificity of the Apt to interact with these cells.

**Fig. 4:**
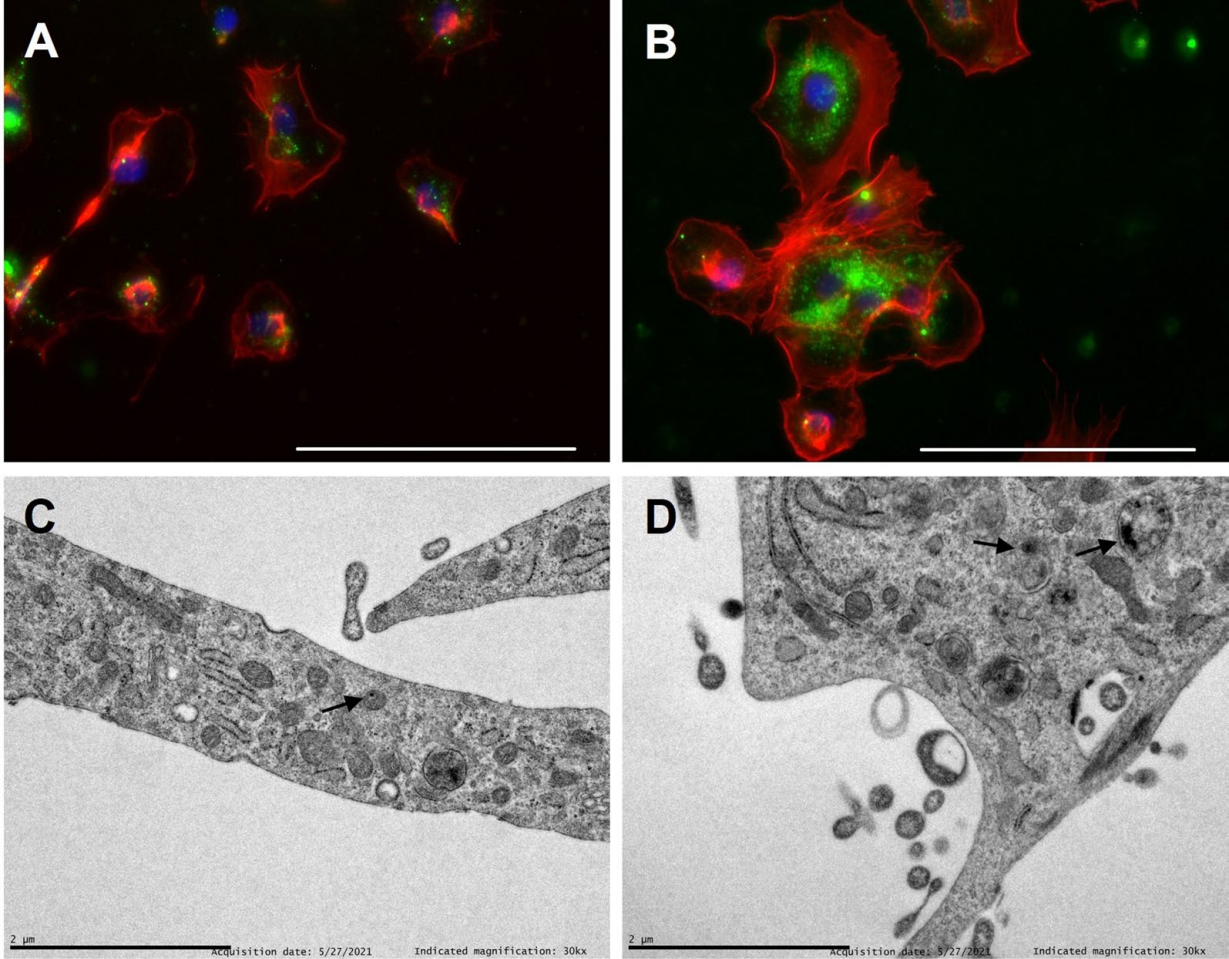
Fluorescent microscopy and transmission electron microscopy images of **A**, **C** LPNPs and **B**, **D** Apt-LPNPs. Nanoparticles are labeled in green, cytoplasm is marked in red and cell nuclei are stained in blue. In electron micrographs LPNPs and Apt-LPNPs are indicated with arrows. Scale bars: Fluorescent microscopy: 20 µm. Electron micrographs: 2 µm

The intracellular localization of NPs was studied both by fluorescence microscopy and transmission electron microscopy. Figure 4A, B shows cells treated with coumain-6 labelled LPNPs (green) and stained with rhodamine-phalloidin to identify cytoplasm (red) and DAPI to visualize nuclei (blue). As it can be gathered from the images, NPs are mainly distributed in the cell cytoplasm. In agreement with the mentioned cytoplasmatic distribution of NPs, TEM micrographs (Fig. 4C, D) also show the nanoparticles (identified as black dots) are localized in the cell cytoplasm and in endocytic vesicles.

### In vitro gene silencing efficiency of LPNPs using primary cells and cell lines

The ability of the developed LPNPs to silence the expression of the target gene, SFRP1, was evaluated in vitro using both primary murine bone marrow mesenchymal stem cells (mMSC) and a well-stablished murine mesenchymal stem cell line (C3H10T1/2). Cells were treated with the nanoparticles suspension or with an equivalent concentration of GapmeR (20 nM) complexed with the commercial transfection agent Dharmafect as positive control. Gene expression was normalized by the housekeeping genes and represented as relative expression to cells treated with LPNPs-Cntrl. Both cell types presented a similar behavior to all the treatments. However, the incorporation of the aptamer to the nanoparticles surface seemed to have more effect on gene silencing in mMSC than on the immortalized cell line.

**Fig. 5:**
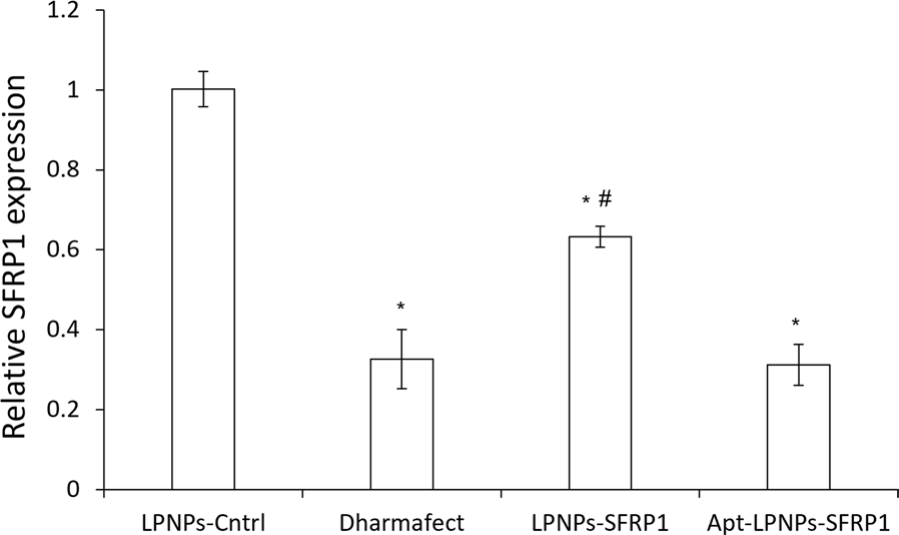
SFRP1 relative expression normalized by LPNPs-Cntrl after housekeeping normalization. (*) denotes statistical significance to LPNPs-Cntrl, (#) denotes statistical significance to the positive control Dharmafect (p < 0.05)

Primary cells treated with SFRP1-Dharmafect complexes showed a significantly lower SFRP1 expression when compared to cells treated with LPNPs-Cntrl (Fig. 5). The treatment with LPNPs-SFRP1 also decreased the expression of SFRP1 but in less extend than the positive control. Nevertheless, when cells were treated with the nanoparticles loaded with SFRP1 and functionalized with the aptamer a higher reduction in SFRP1 expression was detected observing expression levels similar to the ones obtained for the positive control indicative of the adequate LPNPs gene silencing efficiency.

### LPNPs biodistribution

**Fig. 6:**
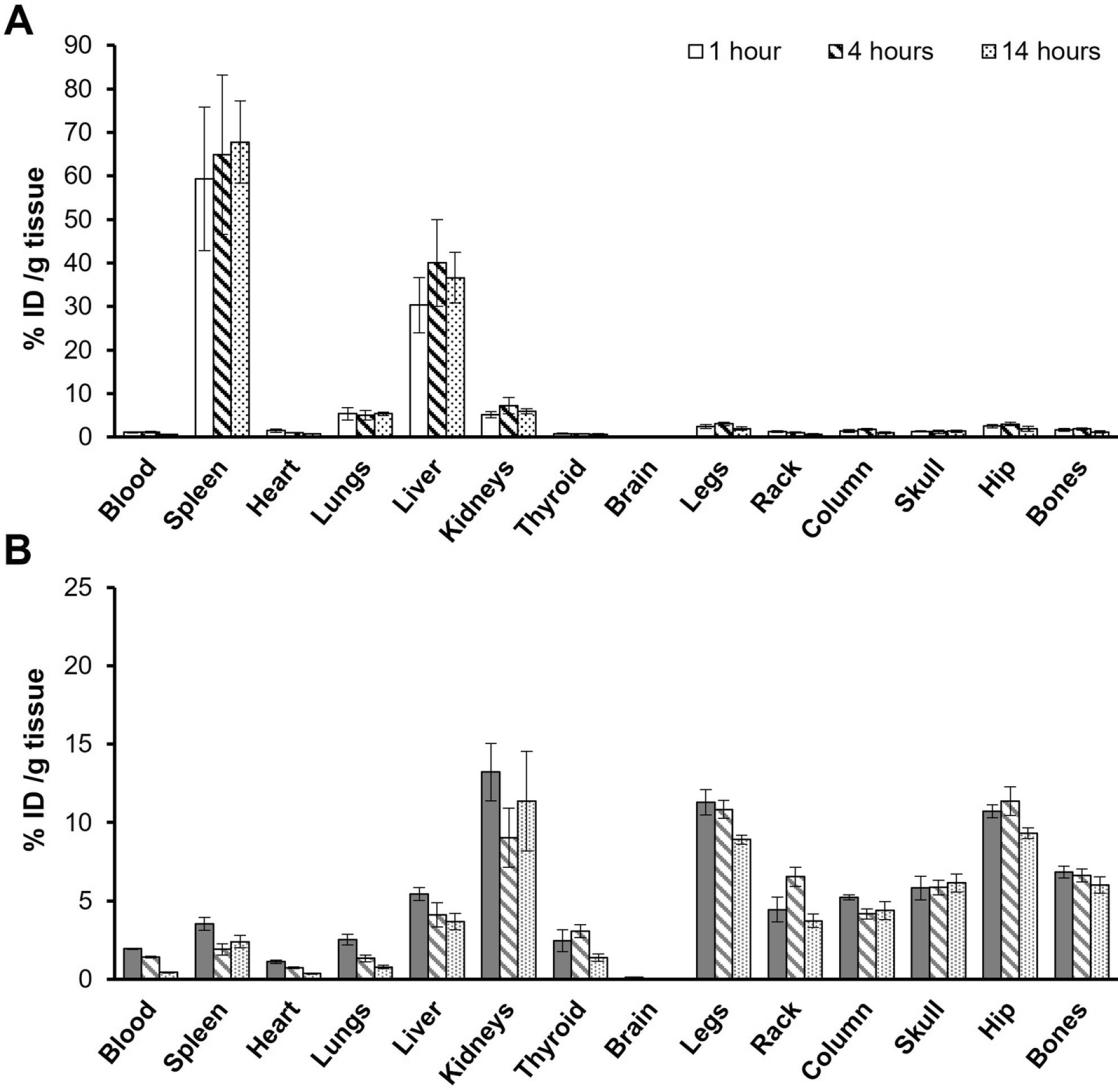
Organ accumulation of **A** Naked LPNPs and **B** Apt-LPNPs after *i.v.* administration using ^99m^Tc radiolabeled NPs. The “bones” value is the average of all the radioactivity measurements of the different bone (n = 4)

Both naked and Apt functionalized LPNPs showed a radiolabeling efficiency higher than 97 ± 1.5%. Furthermore, almost no increase in free ^99m^TcO^−4^ was detected within 24 h after the labeling reaction (< 4%) indicating the good stability of the tagging. Figure 6 shows the percentages of radioactivity, with respect to the injected dose, per gram of tissue (% ID/g) in major organs. Naked LPNPs mainly accumulated in two organs after their i.v. administration, spleen, and liver (Fig. 6A). The highest radioactivity values were detected in the spleen followed by liver, also showing high levels of radioactivity. Further away were the values obtained in lungs and kidneys. It should be noted the radioactivity values in these four organs remained similar during the 14 h of experiment. These results are expected as spleen and liver are highly involved in the clearance of nanoparticles from the bloodstream [42]. The biodistribution profile changed significantly for ^99m^Tc-Apt-LPNPs, where the LPNPs are mostly accumulated in kidneys and bone tissue instead of spleen and liver (Fig. 6B). In this case, the radioactivity observed in liver and spleen was much lower than when naked NPs were injected. Moreover, the high radioactivity levels observed for ^99m^Tc-Apt-LPNPs in the kidneys are expected, and most likely associated to the nanoparticles’ scape of the liver and spleen clearance leading to an increase in renal excretion. These results indicate the aptamer functionalization seems to indeed improve the bone target capacity of the designed nanoparticles.

Finally, it is important to mention that the radioactivity levels in the rest of the tested organs including brain was negligible for both types of nanoparticles, evidencing their inability to cross the blood–brain barrier.

### In vivo osteoinduction capacity of modified NPs: histology, histomorphometry and immunohistochemistry evaluation

**Fig. 7:**
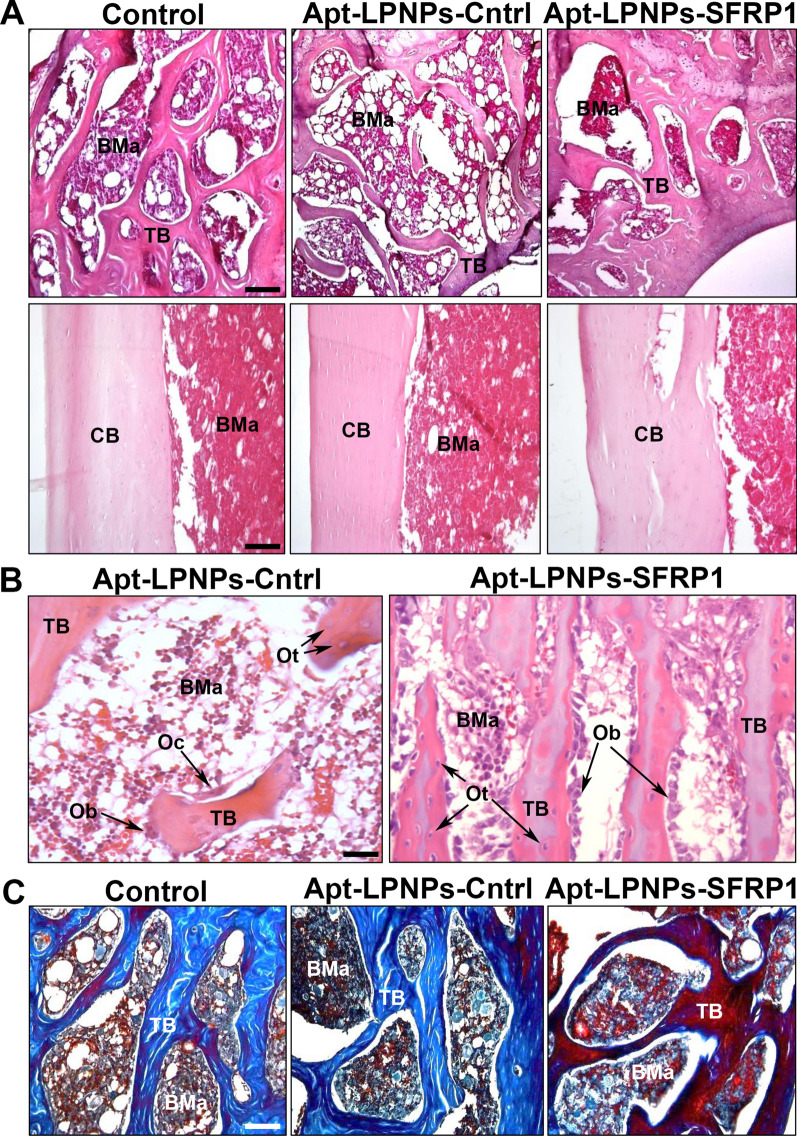
Histology. Representative images showing the trabecular bone microarchitecture (**A**, upper line) and the cortical bone structure (**A**, bottom line) in the three experimental groups (SS, A-A-LPNPs control and A-A-LPNPs). **B** Images at high magnification, showing details of the osteosynthesis cellular activity in the A-A-LPNPs control and A-A-LPNPs groups. Observe the hypertrophic appearance of the osteoblasts (Ob) in the GapmeR-SFRP1 group, indicative of a higher synthesis activity, compared to the scares hypertrophic osteoblasts (Ob) in the A-A-LPNPs control. **C** Representative images with VOF staining technique, showing the mineralization degree in the three experimental groups. Observe the red-brown staining of the trabecular bone in the A-A-LPNPs group, indicative of a bone with a higher degree of mineralization compared to the control groups (SS and A-A-LPNPs control). BMa: bone marrow, CB: cortical bone, Ob: osteoblast, Oc: osteoclast; Ot: osteocytes, TB: trabecular bone. (A and B: H-Er; C: VOF). Scale bars: **A** upper line: 70 µm; bottom line: 60 µm. **B** 40 µm. **C** 50 µm

The histological evaluation of the femurs revealed evident changes in the bone structure and microarchitecture between the different experimental groups (Control, Apt-LPNPs-Cntrl and Apt-LPNPs-SFRP1) (Fig. 7). The hematoxylin-erythrosin staining, showed a more compact trabecular bone and an increase in the thickness of cortical bone, in Apt-LPNPs-SFRP1 compared to the control groups (Control and Apt-LPNPs-Cntrl) (Fig. 7A). Trabecular bone presented a more confluent appearance in Apt-LPNPs-SFRP1, with thicker trabeculae, abundant osteocyte inside the extracellular matrix, and osteoblasts on their surface (Fig. 7A). However, in Control and Apt-LPNPs-Cntrl treated mice, the trabecular bone presented thinner trabeculae and larger medullary cavities containing hematopoietic tissue (Fig. 7B). Furthermore, although unilocular adipocytes were observed in the hematopoietic tissue in animals from all experimental groups, their presence was lower in Apt-LPNPs-SFRP1 (Fig. 7B).

The mineralization analysis by VOF trichrome staining showed the femurs of the Apt-LPNPs-SFRP1 group presented more extensive areas of mineralization than those of the other groups, with a higher degree of bone mineralization, as clearly observed by the intense red coloration both in the trabecular bone and in the cortical bone (Fig. 7C).

**Fig. 8:**
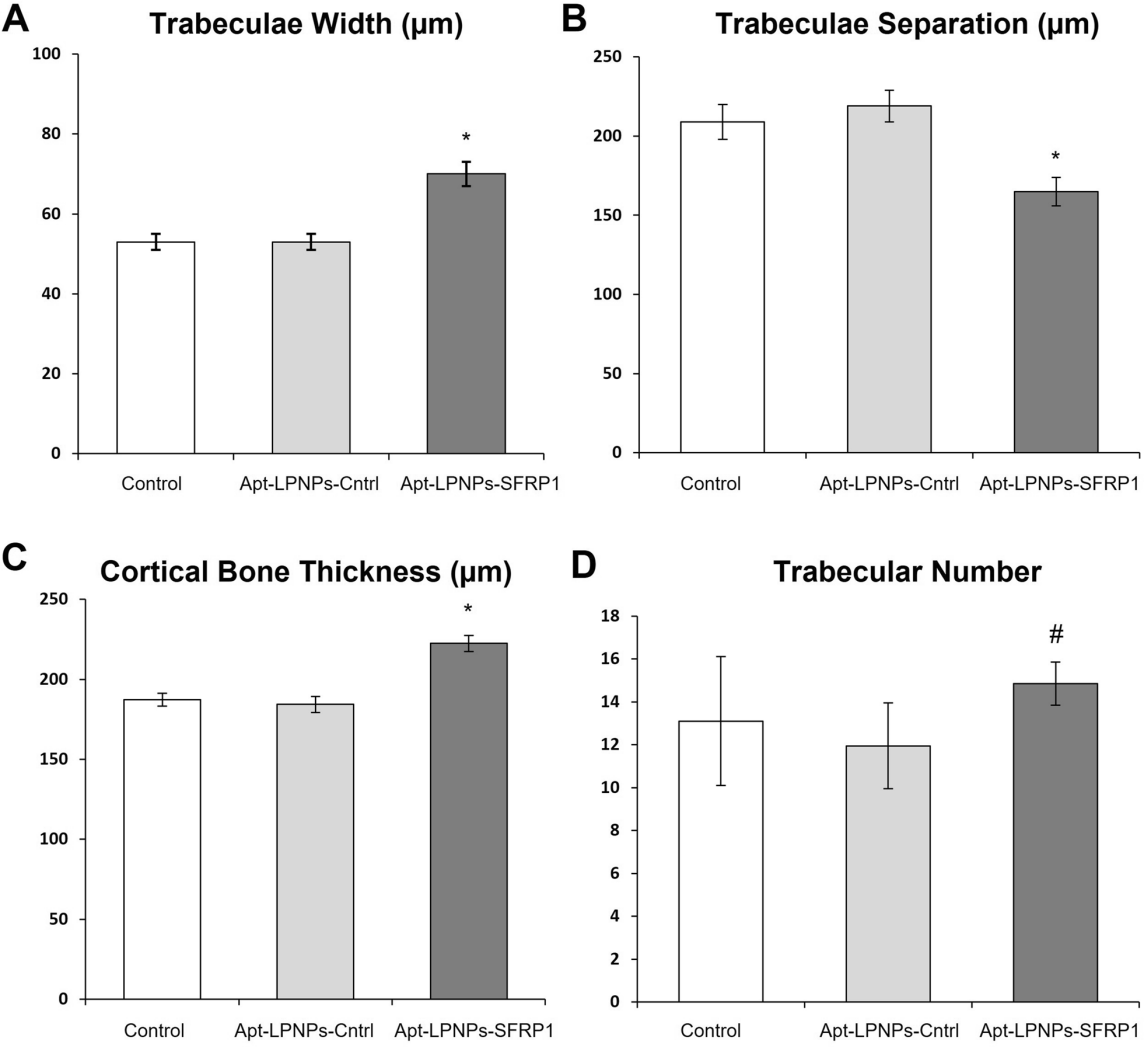
Histomorphometric analysis of femurs from the different experimental groups Control (0.9% saline solution) Apt-LPNPs-Cntrl and Apt-LPNPs-SFRP1.Thickness of the cortical bone (Ct.Wi) (**A**) and number (Tb.N) (**B**), width (Tb.Wi) (**C**) and separation (Tb.Sp) (**D**) of the trabeculae in trabecular bone. Histograms represent mean ± SE values. (*) denotes statistical significance between Control and Apt-LPNPs-Cntrl to Apt-LPNPs-SFRP1, (#) denotes statistical significance between Apt-LPNPs-Cntrl to Apt-LPNPs-SFRP1 (p < 0.005) (n = 6)

The histomorphometric analysis revealed changes in bone structure between the different experimental groups, confirming the histological observations. On one hand, significant differences were found in the thickness of the cortical bone (Ct.Wi), with the Apt-LPNPs-SFRP1 showing a wider cortical bone than Control and Apt-LPNPs-Cntrl groups (Fig. 8A). No significant differences were observed between the control groups (Fig. 8A). Likewise, the microarchitecture of trabecular bone revealed differences in all the evaluated parameters, trabeculae number, trabeculae width and trabeculae space, between the Apt-LPNPs-SFRP1 and control groups (Control and Apt-LPNPs-Cntrl), showing broader trabeculae and in greater numbers (Fig. 8B–D), and therefore more confluent.

**Fig. 9:**
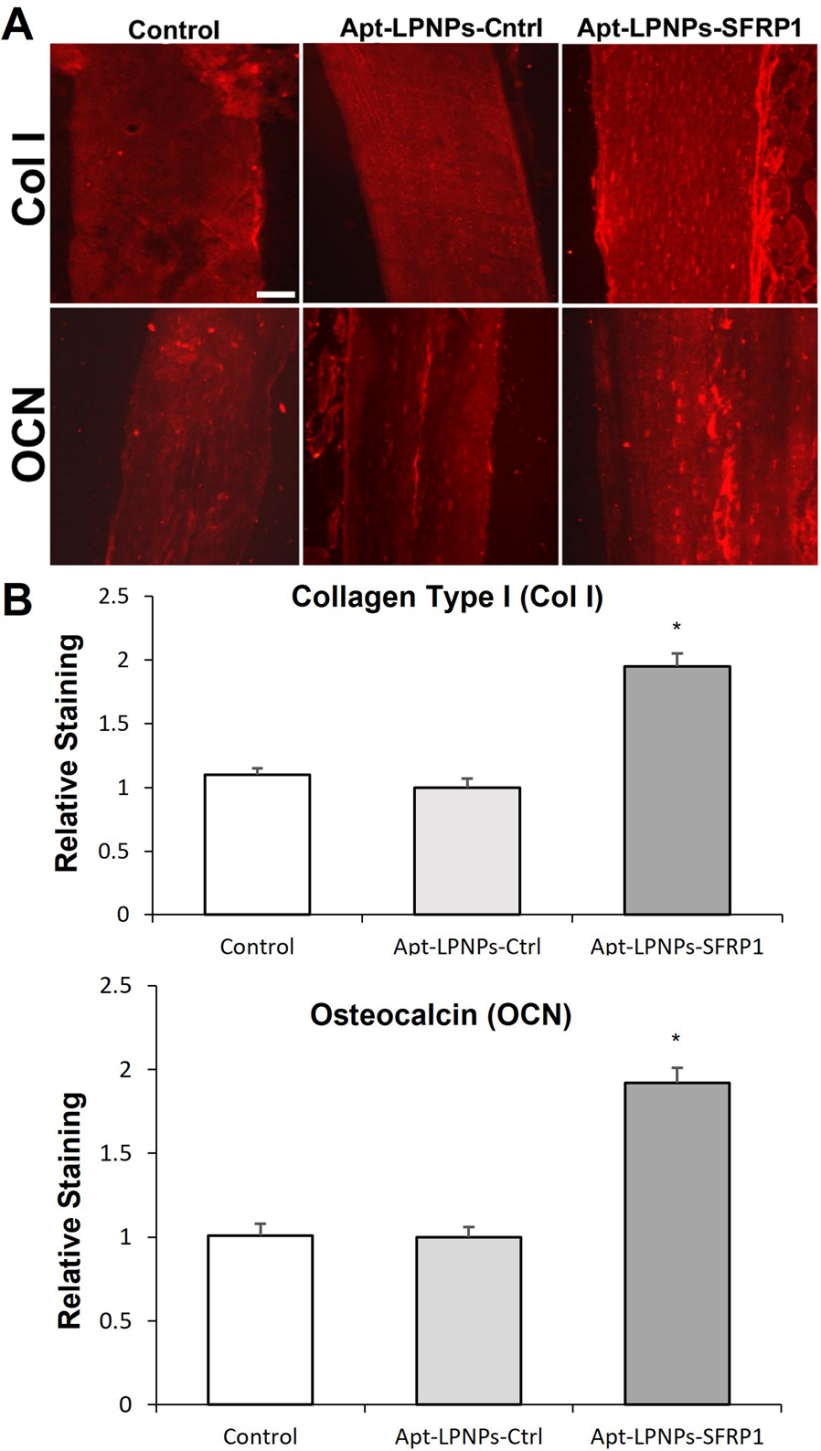
Immunohistochemical analysis of the femur samples from the different experimental groups: Control (0.9% saline solution), Apt-LPNPs-Cntrl and Apt-LPNPs-SFRP1. **A** Semipanoramic images of cortical bone showing the presence of Col I and OCN immunoreactivity. Scale bar: 50 µm. **B** Graphs showing the relative staining intensity of Col I and OCN in the three experimental groups. Histograms represent mean ± SEM values. (*) denotes statistical significance between Control and Apt-LPNPs-Cntrl to Apt-LPNPs-SFRP1 (p < 0.05) (n = 6)

Immunohistochemical analysis of collagen I (Col I) and osteocalcin (OCN), revealed increased immunoreactivity for both markers in the Apt-LPNPs-SFRP1 compared to control groups (Fig. 9A). The histomorphometric analysis of immunofluorescence revealed significantly higher relative staining intensity values in the Apt-LPNPs-SFRP1 group compared to the control groups (Control and Apt-LPNPs-Cntrl) (Fig. 9B).

### Nanoparticles’ toxicity

**Fig. 10:**
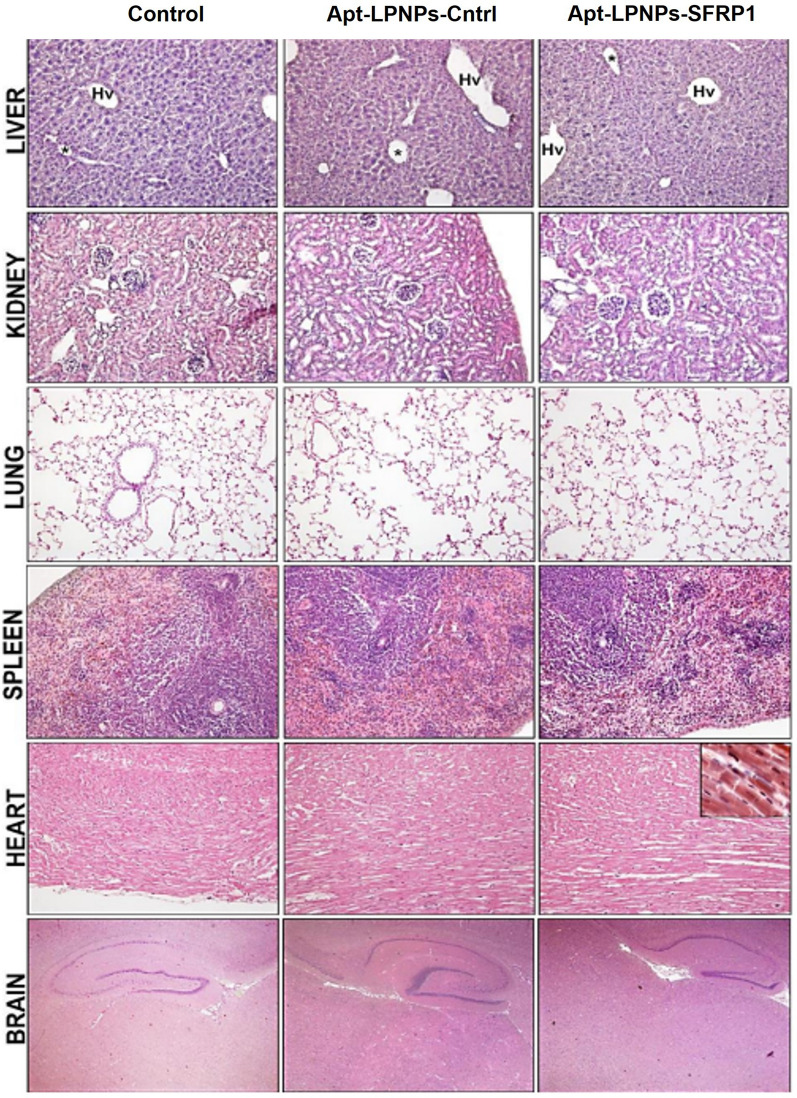
Organs Toxicity. Representative semi-panoramic images in the three experimental groups, Control (0.9% saline solution), Apt-LPNPs-Cntrl and Apt-LPNPs-SFRP1, showing the histological structure of the liver, kidney, lung, spleen, heart and brain. All the organs analyzed showed normal tissue architecture with no apparent changes at the cellular level. Liver: Hv: hepatic vein, PV: portal vein, arrowheads: sinusoid capillaries. Kidney: CR: renal corpuscle, TCP: proximal convoluted tubule, TCD: distal convoluted tubule, arrowhead: renal capsule. Lung: A: artery, B: bronchiole, RB: respiratory bronchiole, AS: alveolar sacs, PA: pulmonary alveoli. Spleen: A: artery, RP: red pulp, WP: white pulp, arrowhead: splenic capsule. Heart: c: cardiomyocyte, E: epicardium, M: myocardium, arrowhead: intercalar disc. Brain: BF: basalforebrain, Hy: hippocampus, TC: telencephalic cortex, V: cerebral ventricles with choroid plexuses. Scale bars: liver, kidney, lung, spleen and heart: 80 µm. Heart insert: 15 µm. Brain: 150 µm

None of the microscopic analysis showed differences in the organs of animals treated with Apt-LPNPs-SFRP1 with respect to the organs of the control groups (Control and Apt-LPNPs-Cntrl). No significant changes were observed in the organs, neither in tissue architecture nor in cell structure with respect to the Control group. The liver showed a normal structure with hepatocytes arranged in cords around the sinusoid capillaries, and normal size and morphology (Fig. 10). The vascular spaces of portal and hepatic systems presented normal size and morphology as well as the bile ducts (Fig. 10). The kidney showed a normal tissue structure both, in cortex and medulla, observing renal corpuscles of normal size and morphology in which the simple flat epithelium of Bowmann's capsule is observed (Fig. 10). The proximal convoluted tubules dominated the parenchyma of renal cortex, showing the characteristic simple cubic epithelium of intense eosinophilic stain and the brush border in the apical domain of epithelial cell (Fig. 10). The distal tubules, of simple cuboidal. The lung showed normal tissue structure without significant changes or signs of fibrosis. Vascular and air spaces of normal size and morphology were observed (Fig. 10). Alveolar and pulmonary sacs presented normal structure without significant changes no thickening of the walls (Fig. 10).

The spleen showed a normal tissue structure without significant changes in cell composition and distribution, clearly observing the differentiation between white pulp and red pulp (Fig. 10). Macrophage density was slightly higher in the groups treated with Apt-LPNPs-SFRP1 compared to Control group, although within normality. The heart presented in all groups a normal histological structure without significant changes. The myocardium showed cardiomyocytes with preserved morphology, showing nuclei in a central position (Fig. 10). The intercalary discs were clearly seen (Fig. 10).

The brain presented a normal histological structure in rostral (Fig. 10) and caudal areas, without significant changes in the distribution of gray and white matter. Ventricles of preserved size and morphology were also observed in which choroid plexuses of normal structure could be identified.

## Discussion

Osteoporosis is a systemic skeletal disease characterized by low bone formation and increased adipose tissue in the bone marrow [43]. Previous studies have shown the inhibition of the SFRP1 expression, gene that encodes the Sfrp1 protein, an antagonist of the Wnt/βcatenin signaling pathway, promotes an increase in the osteogenic potential of MSCs in bone [5, 44]. However, the systemic stimulation of this pathway entails serious side effects as cardiovascular events [45]. The present study was designed to develop adequate lipid-polymer hybrid nanoparticles (LPNPs) suitable for the selective SFRP1 expression inhibition at the bone marrow MSCs. To this end, initially LPNPs were prepared only with DOTAP as the lipid component. However, this composition was not stable when subjected to physiological environments. In a previous study carried in our lab, the tailorable characteristics of LPNPs have been proven showing the surface characteristics can be controlled by the lipid components [30]. Considering our previous study phosphatidylcholine (PC) and a pegylated phospholipid (DSPE-mPEG) were added to the initial LPNPs components. This modification was suitable to avoid the undesirable nanoparticle aggregations likely due to the increase in colloidal stability and hydrophilicity of the nanosystems associated with the presence of PEG while PC enhanced the stability of the lipid layer thanks to its cylindrical geometry [46]. Moreover, the presence of PEG will reduce the LPNPs opsonization and the mononuclear-fagocitic system uptake [47, 48].

To achieve the desired cell-specific silencing, the adequate LPNPs were covalently functionalized with an aptamer specifically designed for murine bone marrow MSCs [21]. This strategy was performed by replacing part of the DSPE-mPEG_2000_ by DSPE-PEG_2000_-MAL similarly as previously reported [29]. This functionalization led to a slight increase in the nanoparticle size and a decrease in zeta potential, similarly, to already reported for aptamer-functionalized hybrid nanoparticles and liposomes [49, 50]. Functionalized nanoparticles were cytocompatible and highly internalized by mMSCs. LPNPs were localized in the cytoplasm of the cells indicating these systems could be internalized by the cells via endocytosis. The inclusion of protamine, a good carrier for nucleic acids could favor the LPNPs internalization and endosomal escape, as has been described [24]. In addition, the lipids of the corona can also be good co-adjuvants to promote cell transfection. In fact, the Apt-LPNPs-SFRP1 produced a reduction of the relative SFRP1 expression in mMSC very similar to that observed with Dharmafect, the positive control that shows a silencing capacity similar to already reported [51].

Moreover, the efficacy of the administration of aptamer functionalized pegylated GapmeR-loaded LPNPs to enhance the osteogenic potential of MSCs in osteoporosis without any toxicity was evaluated. The improvement of the osteoporotic bone formation and the toxicity on the main mice organs were evaluated after the systemic administration of the formulations on a combined ovacteriomized-glucocorticoid osteoporotic mice model previously developed in our lab [32]. We have previously described Sfrp1-silenced MSCs stimulated bone formation in vivo in an ectopic mouse model without showing signs of altered biological effects [6]. However, this strategy of injecting gene- modified MSCs do not ensure the enhancement of bone mass in an OP treatment. To contribute to the development of a drug delivery system with potential clinical application, in the present study we propose a step-forward strategy, producing a MSC-specific stimulation of the Wnt/βcatenin signaling pathway.

To the best of our knowledge, this is the first time that SFPR1-silencing molecules were incorporated into a delivery system and are proposed as bone-targeting nanocarriers for osteoporosis treatment. Knowing the strategic role of NP surface components and properties play on in vivo distribution, the elaborated LPNPs were pegylated and decorated with an aptamer. The selected aptamer was previously used complexed in a PEI-antagomiR-188 system showing the bone-targeting ability mediated by the aptamer [21]. The targeting properties of the Apt-LPNPs were studies in vivo using 25 ng of GapmeR/month, approximately 150 µg of NPs injected in the tail vein of osteoporotic mice. The inclusion of the aptamer on the LPNPs surface indeed demonstrated a modified biodistribution profile with respect to non-modified LPNPs showing an increase in bone and kidney accumulation. Other aptamer-based targeting strategies have been previously reported to promote bone tissue after systemic administration showing a local bone effect. Sema4D siRNA loaded in N‐(2‐hydroxypropyl) methacrylamide (HPMA) systems functionalized with D‐Asp8 injected to ovariectomized mice resulted in an increase of bone volume [52]. More recently, the conjugation of bone marrow stromal cell (BMSC) exosomes with an aptamer to target BMSC stimulated bone regeneration in osteoporotic mice [53].

Osteoporosis, in addition to being characterized by a decrease in mass and an alteration in bone structure and microarchitecture, is also associated with increased bone marrow adipogenesis and decreased osteoblastogenesis [54]. The results of the histological and histomorphometric evaluation evidenced this situation in femur samples from OP mice treated with saline (Control) and with the Apt-LPNPs-Cntrl. However, mice treated with the therapeutic GapmeR (Apt-LPNPs-SFRP1) showed a recovery of the bone structure and microarchitecture, with increased cortical bone thickness and better structural parameters in cancellous bone, as well as a reduction in adipose tissue in the bone marrow. These results provide experimental evidence suggesting the femur of osteoporotic mice treated with the therapeutic GapmeR there suffered an increase in osteosynthesis, probably due to an increase in the osteogenic potential of MSCs induced by the inhibition of the SFRP1 gene. Bodine et al. obtained similar results in a study, in which SFRP1 knockout mice showed increased trabecular bone mass as a result of an increased osteoblast proliferation and differentiation [4]. Also, in a later article they reported that the lack of SFRP1 in mice leads to reduced apoptosis of osteoblasts and osteocytes [55]. It has also been shown that diarylsulfone sulfonamide, a low molecular weight Sfrp1 inhibitor, can bind and inhibit this protein by stimulating the Wnt/β-catenin signaling pathway to increase bone formation [5]. The results obtained from these studies confirmed the role of Sfrp1 in bone formation and suggest that inhibition of the SFRP1 gene may be a potential therapeutic target to increase bone formation. Bone formation is a complex process carried out by osteoblasts that includes the formation of a complex extracellular matrix (ECM) mainly composed of collagen I, proteoglycans, and non-collagenous proteins such as osteocalcin and osteopontin, which is subsequently mineralized by the deposition of hydroxyapatite. ECM also contains adsorbed signaling molecules, such as growth factors and cytokines, of enormous importance in tissue homeostasis [37]. Regarding the formation of the ECM, the immunohistochemical analysis of the femurs of the Apt-LPNPs-SFRP1 group showed greater immunoreactivity for Col I, a marker of early osteogenesis, and for OCN, a marker of late osteogenesis and mineralization. Furthermore, these femurs presented more extensive areas of mineralization than the rest of the experimental groups as observed in the VOF staining. Therefore, the results of the immunohistochemical and bone mineralization analysis agree with the histomorphometric ones, showing the femurs in mice treated with the therapeutic GapmeR show an increase in bone synthesis activity predictably mediated by the increase of the number of osteoblasts. Moreover, the histological study of the main organs did not show any toxic effect of the injected nanoparticles.

In summary, the results obtained in this study show the success of the nanocarriers developed to block the expression of the SFRP1 gene in vivo without any toxic effect. In addition, they agree with the results obtained in previous works [4, 5, 44, 55], demonstrating the inhibition of the expression of SFRP1, a gene that encodes the Sfrp1 protein, increases bone formation, due to an increase in the proliferation and differentiation of osteoblasts.

## Conclusions

The administration of the developed nanoparticles containing an encapsulated GapmeR for silencing SFRP1 and decorated with a specific MSC aptamer proved to be a successful therapy in the treatment of osteoporosis. Mice treated with Apt-LPNPs-SFRP1, showed a more compact trabecular bone and increased cortical bone thickness compared to Apt-LPNPs-Cntrl, with no signs of toxicity. Furthermore, the lipid shell composition of the nanoparticles was optimized to remain stable at different physiological pHs. Additionally, LPNPs were observed to be non-toxic for two cell types present in bone, MSCs and macrophages, in agreement with the histological in vivo observation. On the other hand, the decoration of LPNPs with a MSC-specific aptamer allowed the modification of the nanoparticle biodistribution causing a four-fold increase in the bone nanoparticle accumulation, especially in femur and hip, bones typically affected in osteoporosis and a tenfold decrease in their hepatic and spleen accumulation. All the above suggests the great potential of the decorated nanoparticles for the treatment of osteoporosis. Also, these nanoparticles functionalized with a MSC specific aptamer could have an undiscovered potential in the development of treatments suitable for other bone diseases.

## Supplementary Information

**Additional file 1: Figure S1. **Increase in the optimized NPs uptake after aptamer functionalization in murine MSC (C3H10T1/2) and fibroblasts (BALB/3T3). (*) denotes statistical significance to non-functionalized NPs (LPNPs) p < 0.05 (n = 5).

## Data Availability

The datasets used and analysed during the current study are available from the corresponding author on reasonable request.
