# Supplementary material for: Nanoparticle-mediated selective Sfrp-1 silencing enhances bone density in osteoporotic mice
Source: J Nanobiotechnology. 2022 Oct 29;20:462. doi: 10.1186/s12951-022-01674-5 (PMC9618188; doi:10.1186/s12951-022-01674-5)
Supplement: Supplementary file 1 — Additional file 1: Figure S1. Increase in the optimized NPs uptake after aptamer functionalization in murine MSC (C3H10T1/2) and fibroblasts (BALB/3T3). (*) denotes statistical significance to non-functionalized NPs (LPNPs) p < 0.05 (n = 5). [file 12951_2022_1674_MOESM1_ESM.docx]

**Figure 1**


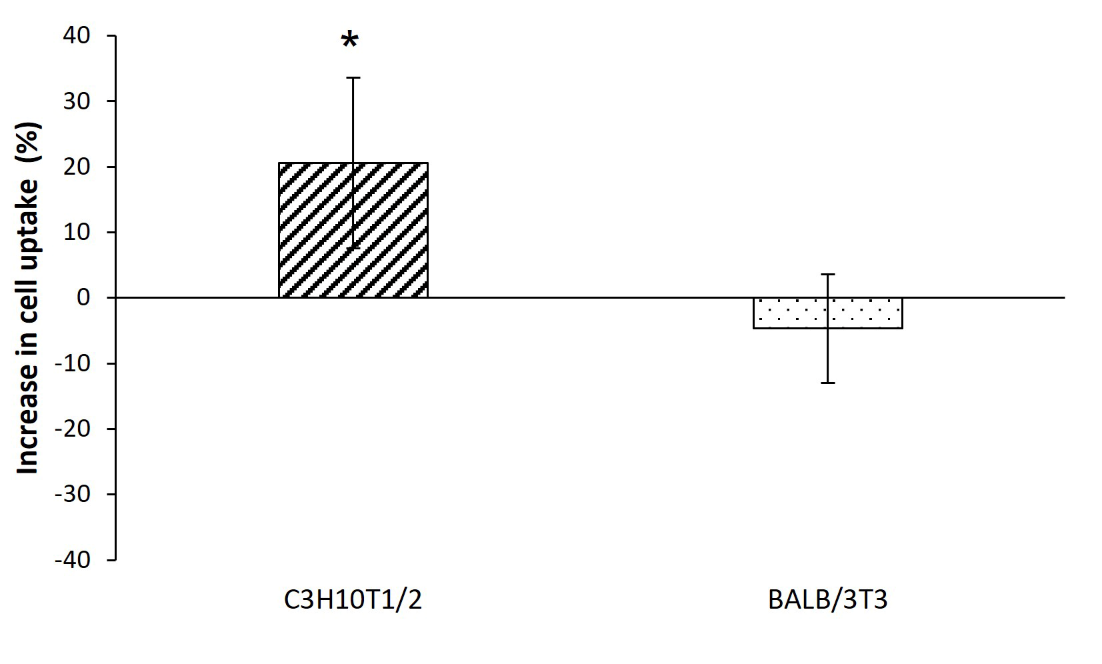


**Additional Figure S1.** Increase in the optimized NPs uptake after aptamer functionalization in murine MSC (C3H10T1/2) and fibroblasts (BALB/3T3). (*) denotes statistical significance to non-functionalized NPs (LPNPs) p < 0.05 (n=5).
